# Lymphatic Targeting of Flubendazole via Long-Chain Fatty Acid Nanoemulsion: Pharmacokinetic Evidence of Chylomicron-Mediated Uptake in Rats

**DOI:** 10.1021/acsomega.6c02880

**Published:** 2026-07-13

**Authors:** Danielle Costa Vargas Redondo, Jéssica Fagionato Masiero, Marcos Cecilio CostaJunior, Emilly Costa de Oliveira, Yasmin da Silva Santos, Nikoletta Fotaki, Raimar Löbenberg, Gabriel Lima Barrosde Araújo, Nádia Araci Bou-Chacra, Leandro Augusto Calixto

**Affiliations:** † Faculty of Pharmaceutical Sciences, 28133 University of São Paulo, São Paulo, São Paulo 05508-010, Brazil; ‡ Department of Exact and Earth Sciences, Institute of Environmental, Chemical and Pharmaceutical Sciences, Federal University of São Paulo, Diadema, São Paulo 04023-062, Brazil; § 1555 University of Bath, Claverton Down, Bath BA2 7AY, Reino Unido; ∥ Faculty of Pharmacy & Pharmaceutical Sciences, University of Alberta, Edmonton, Alberta T6G 2T9, Canada

## Abstract

Flubendazole (FLZ), a benzimidazole anthelmintic with emerging anticancer potential, exhibits poor aqueous solubility, limited oral bioavailability, and negligible spontaneous lymphatic transport. Building on previous work in which a Maisine CC-based FLZ nanoemulsion (FLZ-NE) was developed and shown to prevent the formation of malignant wounds in a murine model, this study provides a mechanistic pharmacokinetic evaluation of its intestinal lymphatic uptake. We investigated FLZ disposition in rats after oral administration of FLZ-NE, with or without cycloheximide pretreatment (FLZ-NE/b) to inhibit chylomicron secretion. Plasma FLZ concentrations were quantified by a validated HPLC-UV method, and concentration-time data were analyzed by two-way ANOVA and noncompartmental analysis. FLZ-NE produced higher systemic exposure (C_max = 2.42 ± 0.34 μg/mL; T_max = 4 h; AUC_0 – t = 15.92 ± 3.78 μg.h/mL) than FLZ-NE/b (C_max = 0.58 ± 0.13 μg/mL; T_max = 2 h; AUC_0 – t = 4.42 ± 1.55 μg.h/mL), corresponding to a 76% reduction in C_max and a 72% reduction in AUC_0 – t under chylomicron blockade. These data indicate that FLZ-NE relies predominantly on intestinal lymphatic transport, in line with the notion that long-chain fatty acid-based, nanoscale formulations can favor chylomicron-mediated uptake. By demonstrating in vivo that this nanoemulsion drives FLZ absorption through a lymphatic component, our findings extend the earlier efficacy-focused malignant wound study with mechanistic pharmacokinetic evidence of lymphatic targeting and support nanoemulsion-based delivery as a rational strategy to improve oral absorption and expand the therapeutic potential of FLZ for lymphatic or metastatic diseases.

## Graphical abstract

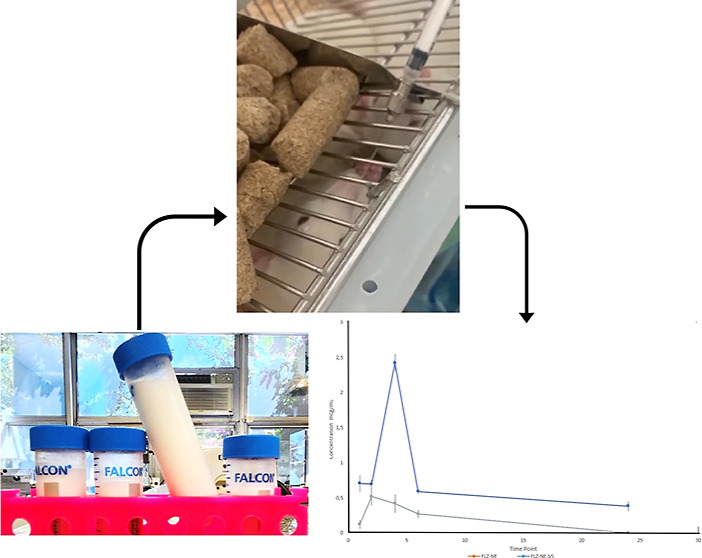

## 1 Introduction

The lymphatic system plays a pivotal role in immune surveillance and lipid transport, but it is also a major pathway for cancer metastasis. Malignant cells can migrate through expanded lymphatic vessels, colonize regional lymph nodes, and subsequently spread to distant organs through lymphatic-to-blood circulation.^1–4^ This dual function—protection and propagation—makes the lymphatic network an essential target for cancer therapy.^1^ Achieving targeted delivery of drug substances to lymphatic tissues can enhance their local concentration, reduce systemic toxicity, and improve therapeutic outcomes, particularly in the treatment of metastatic disease localized in lymph nodes.^5,6^

Intestinal lymphatic transport of orally administered compounds is governed by both the physicochemical properties of the drug and the composition of the formulation. Classically, compounds with high lipophilicity (log *P* > 5), triglyceride solubility above 50 mg/mL, and affinity for long-chain fatty acids are favorably absorbed via the lymphatic route, especially when delivered in colloidal systems with droplet sizes in the 20–500 nm range.^7^ Following lipid digestion, long-chain fatty acids are re-esterified into triglycerides within enterocytes, assembled into chylomicrons with phospholipids, cholesterol, and apolipoprotein B-48, and subsequently secreted into the intestinal lymphatics.^5^ Recent study by Souza et al. (2024)^8^ has demonstrated that lymphatic uptake is attainable even for compounds with relatively low log P values, suggesting that long-chain fatty acids and particle size may exert a stronger influence than lipophilicity alone. These insights underscore the potential of rationally designed lipid-based systems that emulate physiological lipid transport to enhance oral absorption and promote lymphatic targeting while bypassing first-pass hepatic metabolism.

Nanoemulsions have emerged as versatile carriers for lymphatic targeting, owing to their physicochemical stability, nanoscale droplet size, and tunable surface properties.^9^ They can encapsulate lipophilic compounds, facilitate intestinal lymphatic transport, and prolong systemic exposure. When formulated with long-chain fatty acid esters, nanoemulsions are particularly attractive because these lipids stimulate chylomicron formation and enhance the uptake of associated drug substances into the lymphatic system. This strategy is especially relevant in oncology, where oral delivery that preferentially targets lymph nodes offers a noninvasive and potentially more effective approach to controlling metastatic disease.^8–10^

Flubendazole (FLZ), a benzimidazole anthelmintic, has recently gained attention as a potential anticancer agent. In addition to its microtubule-targeting activity, FLZ has been reported to inhibit tumor cell proliferation, induce apoptosis, and modulate oncogenic signaling pathways such as STAT3.^11^ These mechanisms contribute to a multitargeted anticancer profile, positioning FLZ as a promising candidate for drug repositioning.^12–14^ In this context, Yukuyama et al. (2023)^15^ developed and characterized an oral FLZ-loaded nanoemulsion containing Maisine CC as the oil phase. In a murine model, this formulation prevented the development of malignant wounds in 100% of treated mice for up to 80 days, providing strong proof of concept for its antitumor potential. However, despite these promising pharmacodynamic findings, the moderate lipophilicity combined with poor aqueous solubility (0.0344 mg/mL), and unfavorable pharmacokinetic profile of FLZ continue to limit its oral bioavailability and therapeutic reach.^16,17^ Importantly, the previous study by Yukuyama et al. focused mainly on formulation development and in vivo efficacy, leaving the absorption mechanisms and, in particular, the contribution of intestinal lymphatic transport largely unexplored. Thus, our study fills this knowledge gap by providing in vivo evidence of the contribution of intestinal lymphatic transport to FLZ uptake.

Although flubendazole (FLZ) has demonstrated promising anticancer activity in several preclinical studies, its clinical translation remains limited by its poor aqueous solubility, low oral bioavailability, and physicochemical properties that are not typically associated with efficient intestinal lymphatic transport. Nevertheless, emerging evidence suggests that the characteristics of lipid-based nanocarriers, particularly the use of long-chain fatty acids and nanosized droplets, may play a more important role in promoting lymphatic uptake than the intrinsic lipophilicity of the drug itself. Considering the favorable antitumor effects previously observed for the FLZ-loaded nanoemulsion developed by Yukuyama et al. (2023), we hypothesized that this formulation could enhance FLZ absorption through a chylomicron-mediated intestinal lymphatic pathway.

Therefore, the present study was designed to investigate the contribution of intestinal lymphatic transport to the oral absorption of FLZ delivered by a long-chain fatty acid nanoemulsion and to provide mechanistic pharmacokinetic evidence supporting this uptake pathway.

## 2 Materials

FLZ was obtained from Changzhou Yabang—QH Pharmachem CO., LTD (Jiangsu, China). Maisine CC (glyceryl monolinoleate) was acquired from Gattefossé (Sao Paulo, Brazil); Capmul MCM was provided by ABITEC Corp. (Columbus, OH, US); glycerin and polysorbate 80 were purchased from Sigma-Aldrich (Brazil) and Soluplus was donated from BASF (Brazil). Ultrapure water was obtained from a Milli-Q purification system.

FLZ is a benzimidazole carbamate anthelmintic (molecular formula C_16_H_12_FN_3_O_3_; molecular weight ≈313.3 g/mol), bearing a 4-fluorobenzoyl substituent at N-1, a *p*-methoxyphenyl group at C-2, and a methyl carbamate moiety on the imidazole nitrogen. Flubendazole (FLZ) exhibits moderate lipophilicity (logP ≈ 3),^17^ a value that, although indicative of some affinity for lipid phases, remains insufficient to classify it as a compound typically suited for lymphatic absorption. Consequently, formulation strategies are required to overcome this limitation associated with its partition coefficient. Maisine CC (glyceryl monolinoleate) is a mixture predominantly composed of long-chain monoglycerides derived from linoleic acid (C18:2), with a minor proportion of di- and triglycerides. This composition confers high lipophilicity and low hydrophilicity (low HLB), favoring the dissolution of hydrophobic drugs in the oil phase and the formation of stable nanostructures under appropriate emulsification conditions. From a biological standpoint, long-chain lipids rich in linoleic acid are known to stimulate chylomicron biogenesis in enterocytes, promoting drug association with the lipoprotein fraction and subsequent transport via the intestinal lymphatic pathway.^5,7,19^ Thus, the selection of Maisine CC as the oil phase was motivated both by its ability to solubilize flubendazole and by its potential to favor chylomicron-mediated absorption. The chemical structure of FLZ and Maisine CC is depicted in Figure 1.

**Figure 1:**
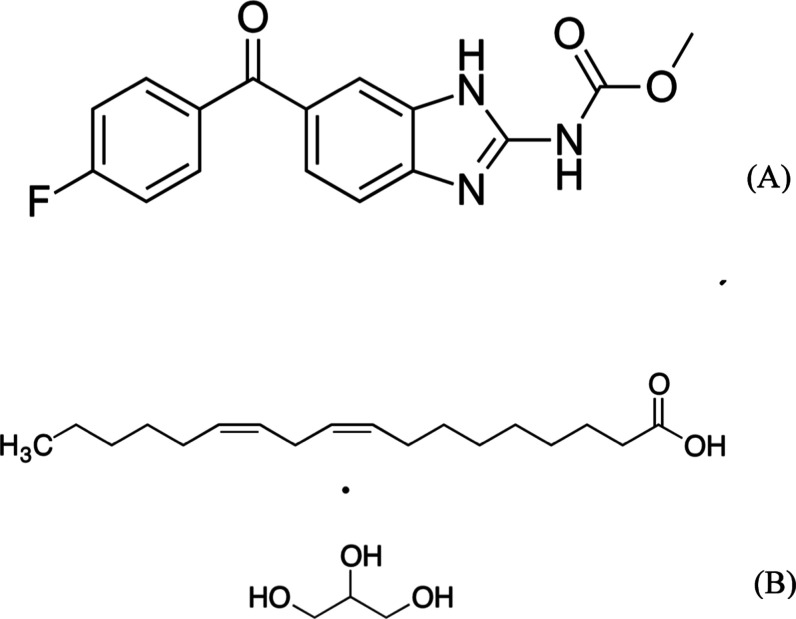
Chemical structure of flubendazole (A) and schematic structure of glyceryl monolinoleate, the main component of Maisine CC (B), illustrating the long-chain unsaturated fatty acid moiety (C18:2) linked to glycerol.

## 3 Method

### 3.1 FLZ-NE Preparation

The oral flubendazole nanoemulsion (FLZ-NE) used in this study was previously developed and optimized by Yukuyama et al. (2023),^15^ who incorporated FLZ into a long- and medium-chain fatty acid nanoemulsion using the d-phase emulsification method, with Maisine CC as the oil phase (Table S1) and polysorbate 80 (4.0% w/w) as the surfactant. Maisine CC was selected based on its content of long-chain monoacylglycerides, which are efficiently re-esterified and incorporated into chylomicrons in the enterocytes, a prerequisite for intestinal lymphatic transport of associated drug substances. To investigate whether FLZ-NE enables lymphatic delivery via chylomicron-mediated transport, it was compared with a nanoemulsion administered under chylomicron flow inhibition (FLZ-NE/b). Both formulations contained FLZ at 5 mg/kg.

### 3.2 Physicochemical Characterization of FLZ-NE

The average hydrodynamic diameter (AHD) and polydispersity index (PdI) of FLZ-NE were determined by dynamic light scattering (DLS) using a Zetasizer Nano ZS90 (Malvern Instruments, Malvern, UK), after appropriate dilution with ultrapure Milli-Q water. The zeta potential was measured based on the electrophoretic mobility of dispersed particles under controlled conductivity conditions (purified water with conductivity adjusted to 50 μS cm^–1^ using 0.2% (w/v) NaCl, *n* = 3), and values were calculated using the Smoluchowski equation.

FLZ quantification and encapsulation efficiency were determined using the same procedures previously established by Yukuyama et al. (2023). FLZ-NE samples were centrifuged with Amicon Ultra filters (50 kDa) at 5000 rpm for 10 min at 25 °C, and the supernatant containing unencapsulated FLZ was diluted in acetonitrile and analyzed by UV spectrophotometry at 310 nm. These procedures were reproduced without modification to ensure methodological consistency.

### 3.3 Animals and Experimental Design

The study protocol was approved by the Institutional Animal Care and Use Committee (protocol no. 609) and complied with the “Principles of Laboratory Animal Care” (National Society for Medical Research) and the NIH “Guide for the Care and Use of Laboratory Animals” (publication no. 86-23, revised 1996).

Male Wistar rats (200–250 g) were maintained under controlled environmental conditions (25 ± 5 °C, 60 ± 5% relative humidity, 12 h light/dark cycle) with ad libitum access to food and water. Rats were fasted overnight before oral administration of the formulations. Random allocation was used, with up to four animals per cage prior to sampling.

### 3.4 Experimental Groups

Animals were randomized into two experimental groups (*n* = 30 per group): FLZ-NE and FLZ-NE/b. Each rat received 5 mL/kg of the respective formulation (equivalent to 1 mg/kg FLZ). In the FLZ-NE/b group, rats were pretreated with cycloheximide (5 mg/kg, intraperitoneally, in saline) to block chylomicron secretion prior to FLZ-NE administration.

For pharmacokinetic studies, plasma samples were collected at 1, 2, 4, 6, and 24 h postdose. Six animals were used per time point from each group (total = 30 per group). All rats were trained for voluntary oral ingestion using a palatable vehicle 1 week before the experiments.^18^ Conditioning consisted of daily afternoon administration of a vanilla–sugar solution (2 g of sugar and 200 μL of vanilla essence dissolved in water), provided via pipet or gavage needle (Supporting Information Video S1).

### 3.5 Biological Material Collection

At each designated time point (1, 2, 4, 6, and 24 h postdose), animals were anesthetized with isoflurane and euthanized. Blood was collected into heparinized tubes and centrifuged at 3000 rpm for 15 min to obtain plasma. The resulting plasma fraction was transferred to clean tubes and stored at −80 °C until HPLC-UV analysis.

A 250 μL aliquot of plasma was mixed with 250 μL of acetonitrile (1:1 v/v) for protein precipitation, followed by a second centrifugation under identical conditions. The upper clear phase was carefully collected, transferred to HPLC vials, and analyzed.

### 3.6 Sample Processing and Quantification

FLZ concentrations in plasma were determined using a validated HPLC-UV method (λ = 246 nm) on a C18 column, employing acetonitrile/potassium phosphate buffer (40:60 v/v) as the mobile phase at a flow rate of 1.0 mL/min.

### 3.7 Statistical Analysis

Data from the pharmacokinetic study are presented as mean ± standard deviation (SD) for plasma flubendazole (FLZ) concentrations at each sampling time point and for each formulation (*n* = 6 rats per formulation and per time). Because a terminal sampling design was used, each animal contributed a single plasma sample at one time point. Plasma FLZ concentrations were first evaluated for homogeneity of variances using Levene’s test. Normality was assessed by visual inspection of residuals (normal probability plots and residual-versus-fitted plots) from the analysis of variance models. When these assumptions appeared reasonably satisfied, parametric methods were applied; otherwise, the results were interpreted descriptively.

To assess the effect of the formulation on systemic exposure, mean plasma concentrations at each sampling time were compared between FLZ-NE and FLZ-NE/b using two-way analysis of variance (ANOVA) with “formulation” (2 levels) and “time” (5 levels) as fixed factors and the formulation × time interaction term, treating the observations as independent across animals and time points.

Because only a single plasma sample was obtained per animal, noncompartmental pharmacokinetic (PK) parameters (Cmax, Tmax, AUC0-t) were derived from the mean concentration–time curves for each formulation and are reported as summary descriptors to support mechanistic interpretation. All statistical analyses were performed using Minitab (Minitab LLC, State College, PA, USA), and a p-value <0.05 (α = 0.05) was adopted as the threshold for statistical significance in the ANOVA.

## 4 Results and Discussion

### 4.1 Physicochemical Characterization of FLZ-NE

Table 1 summarizes the physicochemical characteristics of the prepared FLZ-NE formulations for animal studies. The FLZ-NE demonstrated a high encapsulation efficiency of 98.3 ± 2.5%, confirming successful incorporation of the lipophilic drug substance into the lipid core. Dynamic light scattering analysis revealed an average hydrodynamic diameter (AHD) of 85.5 ± 4.1 nm, positioning the formulation within the optimal size range for lymphatic transport (10–100 nm).^6,19^ The colloidal system exhibited moderate size heterogeneity, as indicated by a PdI of 0.350 ± 0.080, and presented a monomodal distribution. Although values below 0.2 represent ideal monodispersity and those under 0.25 suggest good physical stability,^20,21^ PdI values up to 0.4 remain compatible with acceptable uniformity,.^20^ Furthermore, the formulation’s zeta potential of −24.5 ± 3.2 mV reflects a moderate negative surface charge, contributing to colloidal stability through electrostatic repulsion, a characteristic consistent with stable systems when absolute values exceed ±20 mV.^21^

**Table 1:** Physicochemical Characterization of the FLZ-NE: FLZ Concentration (mg/g), Encapsulation Efficiency (EE %), Average Hydrodynamic Diameter (AHD), Polydispersity Index (PdI), and Zeta Potential (ZP)^a^

sample	mg/kg	EE % (m/v)	AHD (nm)	PdI	ZP (mV)
FLZ-NE	5.0	98.30 ± 2.50	85.5 ± 4.1	0.35 ± 0.10	–24.5 ± 3.2

a Theoretical FLZ concentration (mg/kg), encapsulation efficiency (EE % m/v), average hydrodynamic diameter (AHD), polydispersity index (PdI), and zeta potential (ZP). Data are presented as mean ± standard deviation (*n* = 3). Abbreviation: FLZ-NE: flubendazole-loaded nanoemulsion.

### 4.2 Pharmacokinetic Profile of FLZ Formulations and Mechanistic Evidence of Chylomicron-Mediated Intestinal Lymphatic Uptake

The method for the identification and quantification of flubendazole in plasma suggested high selectivity, as illustrated by Figure 2. Quantification was performed based on the main peak, which emerged at 3.89 min and represented the largest chromatographic area. Calibration curves were linear across 0.1–10 μg/mL (*r* ^2^ > 0.999), with accuracy and precision values within accepted bioanalytical validation criteria. Although secondary peaks were observed, the specificity of the method was not compromised. Thus, the method was established as suitable for the pharmacokinetic studies.

**Figure 2:**
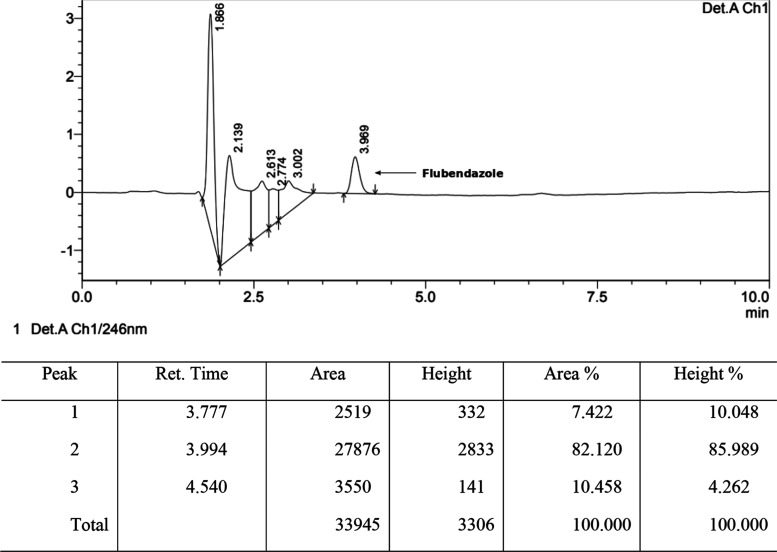
Chromatographic parameters (retention time, peak area, and peak height) from the HPLC quantification of FLZ.

Chromatographic profile and quantification data from the HPLC analysis of a FLZ standard. The table summarizes the retention time, absolute and relative area, and absolute and relative height for each detected peak. The results confirm the identity and purity of the FLZ peak (Peak 2 at 3.994 min), which is the major constituent in the analysis.

Oral administration of the two FLZ formulations in rats produced distinct plasma concentration–time profiles, reflecting the impact of nanoemulsion delivery and chylomicron blockade on drug absorption. Visual inspection of the concentration–time curves (Figure 3) shows higher and more sustained plasma levels for FLZ-NE, whereas cycloheximide pretreatment (FLZ-NE/b) markedly attenuated systemic exposure. To compare formulations and support mechanistic interpretation, plasma FLZ concentrations were subjected to inferential statistical analysis.

**Figure 3:**
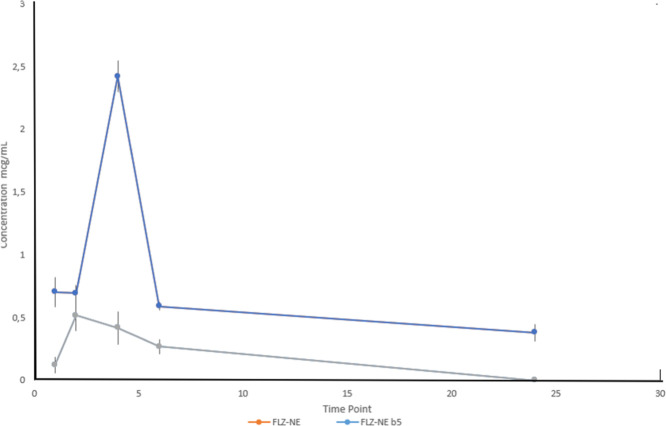
Mean plasma concentration–time profile of flubendazole in rats (*n* = 6) following oral administration of FLZ-NE and FLZ-NE/b formulations. Samples were collected at 1, 2, 4, 6, and 24 h postadministration.

Mean plasma concentration–time profiles (*n* = 6) following oral administration of flubendazole-loaded nanoemulsion (FLZ-NE) and the nanoemulsion administered under chylomicron flow inhibition (FLZ-NE/b). Blood samples were collected at predetermined time points (1, 2, 4, 6, and 24 h) postdose. The arrow highlights the difference in Tmax, indicating a peak at 4 h for FLZ-NE and illustrating the enhanced absorption rate achieved with the nanoemulsion system.

Figure 4 shows that the assumptions required for the application of parametric methods were reasonably satisfied. The normal probability plot of residuals indicates an approximately linear pattern, with no marked deviations at the extremes, supporting the assumption of normality for plasma FLZ concentrations. In parallel, the test for equality of variances does not reveal significant heteroscedasticity between the experimental factors, suggesting that variability is comparable across formulations and time points. Together, these results justify the use of two-way ANOVA to compare FLZ-NE and FLZ-NE/b and to interpret the impact of chylomicron blockade on systemic exposure.

**Figure 4:**
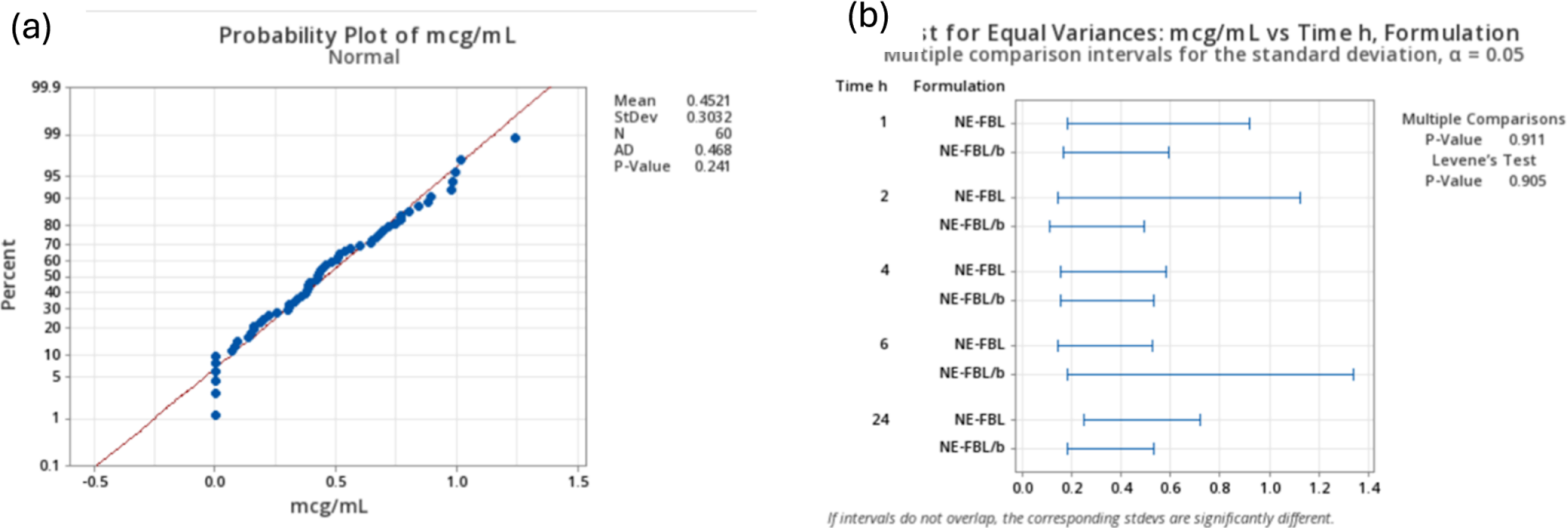
Normal probability plot of flubendazole plasma concentrations (μg/mL) (a) and test for equal of variances (b) across experimental factors.

It is well established in the literature that flubendazole, in its free form, is not transported to the lymphatic system due to its low lipid solubility and limited capacity to associate with chylomicrons, which requires the use of lipid nanocarriers to overcome this physiological barrier, promote association with lipoproteins, and enable intestinal lymphatic targeting.^22–24^

FLZ-NE: flubendazole-loaded nanoemulsion FLZ-NE/b: nanoemulsion administered under chylomicron flow inhibition.

Formulation levels: FLZ-NE (flubendazole nanoemulsion), FLZ-NE/b (flubendazole nanoemulsion with lymphatic blockade). DF: degree of freedom; Adjusted SS: adjusted sum of square; Adjusted MS: adjusted mean square.

A two-way analysis of variance (ANOVA) (Table 2) was conducted to evaluate the effects of formulation (FLZ-NE and FLZ-NE/b) and sampling time (1, 2, 4, 6, and 24 h) on plasma flubendazole (FLZ) concentrations (Table 2). The analysis demonstrated a highly significant main effect for the formulation factor (F_4,50_ = 31.23; *p* < 0.001; ∝ = 0.05), indicating that the delivery system (FLZ-NE versus FLZ-NE/b) exerts a substantial influence on the drug’s systemic availability.

**Table 2:** Two-Way ANOVA for Plasma Flubendazole (FLZ) Concentrations (Factors: Time and Formulation)

source	DF	adjusted SS	adjusted MS	*F*-value	*p*-value
time (h)	4	0.3233	0.08083	1.36	0.261
formulation	1	1.8536	1.85364	31.23	0.000
time (h) × formulation	4	0.2805	0.07013	1.18	0.330
error	50	6.7639	0.05936		

Conversely, the main effect of sampling time was not statistically significant (F_4,50_ = 1.36; *p* = 0.261; ∝ = 0.05), suggesting that temporal variations did not serve as a primary driver of concentration variance within the evaluated intervals. Furthermore, no significant interaction between formulation and time was detected (F_4,50_ = 1.18; *p* = 0.330; ∝ = 0.05). The absence of a significant interaction indicates that the performance differential between the two formulations remained consistent throughout the 24 h study period, rather than exhibiting significant fluctuations at specific time points.

The noncompartmental pharmacokinetic (PK) parameters further quantify the impact of the delivery system and the role of the intestinal lymphatic route (Table 3). In contrast, when the lymphatic pathway was blocked (FLZ-NE/b), systemic exposure was markedly attenuated. The *C*_max_ and AUC_0‑*t*_ for the FLZ-NE/b group decreased significantly to 0.58 ± 0.13 μg mL^–1^and 4.42 ± 1.55 μg mL^–1^ respectively (p < 0.01; α = 0.05).

**Table 3:** Non-Compartmental Pharmacokinetic Parameters of Flubendazole (FLZ) after Oral Administration of the Different Formulations (Mean ± SD, *n* = 6)^a^

formulation	*C*_max_ (μg·mL^–1^)	*T*_max_ (h)	AUC_0–t_ (μg·h·mL^–1^)
FLZ-NE	2.42 ± 0.34	4.0	15.92 ± 3.78
FLZ-NE/b	0.58 ± 0.13	2.0	4.42 ± 1.55

a FLZ-NE: flubendazole nanoemulsion; FLZ-NE/b: nanoemulsion administered under chylomicron flow inhibition; *C*_max_: he highest concentration of a drug in the blood; *T*_max_: the time it takes for a drug to reach the maximum concentration (*C*_max_); AUC: total drug absorption over time.

Specifically, the lymphatic blockade resulted in a 76% reduction in C_max and a 72% reduction in the total extent of absorption (AUC). These data suggest that the systemic availability of flubendazole administered via nanoemulsion is strongly influenced by intestinal lymphatic transport via chylomicron-mediated pathways and are consistent with the notion that such transport can help reduce the impact of first-pass hepatic metabolism.

These findings indicate that lymphatic targeting can be strategically manipulated through formulation design and modulation of chylomicron flow, which is particularly relevant for molecules intended to act within lymphatic tissues or at metastatic sites located in lymph nodes. Future studies should determine whether this strategy translates into increased FLZ accumulation in disease-affected lymph nodes (e.g., *Echinococcus* spp. infection or metastatic cancer), potentially enabling more localized therapy with reduced systemic toxicity.

Our study suggests that the nanoemulsion (FLZ-NE) relies predominantly on the intestinal lymphatic route, as evidenced by the chylomicron flow inhibition model. This observation is consistent with emerging evidence that the lipid composition and nanoscale architecture of the delivery system may exert a greater influence on intestinal lymphatic uptake than the inherent physicochemical properties of the active compound.^8^ For instance, Santos et al. (2024) reported substantial lymphatic uptake of hydroxymethylnitrofurazone (NFOH, log P 0.74) via nanostructured lipid carriers, challenging the notion that high drug lipophilicity is mandatory. In this context, the nanometric size of FLZ-NE (∼85.5 nm) and the use of a long-chain monoacylglyceride-rich oil (Maisine CC) are consistent with formulations known to favor chylomicron formation and lymphatic transport, which may help mitigate the limitations associated with flubendazole’s moderate partition coefficient and support its repositioning for diseases involving lymphatic or nodal involvement.

## 5 Conclusion

Building directly on the previously developed FLZ nanoemulsion, this study suggests that the original long-chain fatty acid nanoemulsion (FLZ-NE) provides higher systemic exposure of flubendazole than its chylomicron-blocked variant (FLZ-NE/b) in rats, as evidenced by increased C_max and AUC_0-t. Cycloheximide-induced inhibition of chylomicron formation markedly reduced FLZ exposure from FLZ-NE/b, supporting a chylomicron-mediated lymphatic contribution to FLZ absorption from this nanoemulsion. By adding mechanistic pharmacokinetic evidence of lymphatic involvement in FLZ uptake, these findings extend the earlier formulation and malignant wound efficacy study and support long-chain fatty acid nanoemulsions as a rational approach for oral delivery of repositioned anticancer agents targeting lymphatic and metastatic disease.

## Supplementary Material





## Notes

^‑^Table S1. Flubendazole solubility in liquid lipids (Yukuyama, 2023) (PDF).
- Video S1. Voluntary oral ingestion of the formulation in the in vivo study. A mp4 file showing the administration procedure used in the animal experiment (MP4).
