# Supplementary material for: Lymphatic Targeting of Flubendazole via Long-Chain Fatty Acid Nanoemulsion: Pharmacokinetic Evidence of Chylomicron-Mediated Uptake in Rats
Source: ACS Omega. 2026 Jul 13;11(29):43763–9. doi: 10.1021/acsomega.6c02880 (PMC13425482; doi:10.1021/acsomega.6c02880)
Supplement: Supplementary file 1 [file ao6c02880_si_001.pdf]

Supporting Information for

Lymphatic Targeting of Flubendazole via Long-Chain Fatty Acid Nanoemulsion: Pharmacokinetic Evidence of Chylomicron-Mediated Uptake in Rats

Danielle Costa Vargas Redondo[a]; Jéssica Fagionato Masiero[a]; Marcos Cecilio Costa Junior[b]; Emilly Costa de Oliveira[a]; Yasmin da Silva Santos[a], Nikoletta Fotaki[c]; Raimar Löbenberg[d]; Gabriel Lima Barros de Araújo[a]; Nádia Araci Bou-Chacra[a]*; Leandro Augusto Calixto[b].*

Video S1. Voluntary oral ingestion of the formulation in the in vivo study. A mp4 file showing the administration procedure used in the animal experiment.

Table S1. Flubendazole solubility in liquid lipids (Yukuyama, 2023)

Samples	Flubendazole[a] (mg)	Lipid[a] (g)	Solubilization (0 day)
Maisine CC	5.06	15.01	Yes[b]
Labrafac Lip. WC1349	5.25	24.86	Yes
Miglyol 812	5.32	33.32	Yes
Captex 8000	5.78	34.39	No
Captex 300	5.05	38.66	No
Captex 355	5.65	42.56	No

[a] total amount of drug / lipid used in the solubility test.

[b] no presence of precipitate during 14 days visual analysis.

Reference:

Yukuyama MN. Repositioning study of flubendazole for treating lung cancer and meningoencephalitis using an oral lipid nanosystem. [São Paulo]: Universidade de São Paulo; 2023. doi:10.11606/T.9.2023.tde-15052023-112430
